# Anemia and clinical outcomes in patients with non-dialysis dependent or dialysis dependent severe chronic kidney disease: a Danish population-based study

**DOI:** 10.1007/s40620-019-00652-9

**Published:** 2019-10-05

**Authors:** Gunnar Toft, Uffe Heide-Jørgensen, Heleen van Haalen, Glen James, Katarina Hedman, Henrik Birn, Christian F. Christiansen, Reimar W. Thomsen

**Affiliations:** 1 Department of Clinical Epidemiology, Aarhus University Hospital, Aarhus, Denmark; 2 AstraZeneca, Gothenburg, Sweden; 3 AstraZeneca, Cambridge, UK; 4 Department of Renal Medicine, Aarhus University Hospital, Aarhus, Denmark; 5 Department of Biomedicine, Aarhus University, Aarhus, Denmark

**Keywords:** Chronic kidney disease, Anemia, Cardiovascular events, Dialysis, Mortality

## Abstract

**Background:**

Routine clinical evidence is limited on clinical outcomes associated with anemia in patients with severe chronic kidney disease (CKD).

**Methods:**

We linked population-based medical databases to identify individuals with severe CKD (eGFR < 30 mL/min/1.73 m^2^) in Northern Denmark from 2000 to 2016, including prevalent patients as of 1 January 2009 or incident patients hereafter into the study. We classified patients as non-anemic (≥ 12/≥ 13 g/dl hemoglobin (Hgb) in women/men), anemia grade 1 (10–12/13 g/dl Hgb in women/men), 2 (8–10 g/dl Hgb), and 3+ (< 8 g/dl Hgb), allowing persons to contribute with patient profiles and risk time in consecutively more severe anemia grade cohorts. Patients were stratified by dialysis status and followed for clinical outcomes.

**Results:**

We identified 16,972 CKD patients contributing with a total of 28,510 anemia patient profiles, of which 3594 had dialysis dependent (DD) and 24,916 had non-dialysis dependent (NDD) severe CKD. Overall, 14% had no anemia, 35% grade 1 anemia, 44% grade 2 anemia and 17% grade 3+ anemia. Compared to patients with no anemia, adjusted hazard ratios (HRs) for NDD patients with grade 3+ anemia were elevated for incident dialysis (1.91, 95% CI 1.61–2.26), any acute hospitalization (1.74, 95% CI 1.57–1.93), all-cause death (1.82, 95% CI 1.70–1.94), and MACE (1.14, 95% CI 1.02–1.26). Similar HRs were observed among DD patients.

**Conclusions:**

Among NDD or DD patients with severe CKD, presence and severity of anemia were associated with increased risks of incident dialysis for NDD patients and with acute hospitalizations, death and MACE for all patients.

**Electronic supplementary material:**

The online version of this article (10.1007/s40620-019-00652-9) contains supplementary material, which is available to authorized users.

## Introduction

Anemia is common in patients with chronic kidney disease (CKD) and prevalence increases with CKD severity [1]. Renal erythropoietin-producing cells sense low tissue oxygen tension and respond by the production of erythropoietin, a hormone that stimulates red blood cell production [2]. CKD leads to a disruption of this process, erythropoietin deficiency and subsequent anemia, characterized by lower than normal number of circulating red blood cells or decreased levels of hemoglobin (Hgb) [3]. Other possible causes of anemia include blood loss, iron deficiency, inflammation and accumulation of uremic toxins [4]. Prevalent anemia in CKD has been associated with cognitive impairment, sleep disturbances, CKD progression, cardiovascular disease and higher mortality in mostly older studies and in selected populations [5–13], whereas contemporary unselected population-based data on clinical outcomes associated with anemia are scarce. Treatment options for anemia include iron (oral and intravenous), erythropoietin stimulating agents (ESAs) and red blood cell transfusion to restore hemoglobin levels. Concerns have been raised regarding the cardiovascular safety of treating anemia to higher Hgb levels, in particular when using ESAs to target Hgb levels > 12 g/dl [14–16]. This has resulted in a change of anemia management practices since 2011, with generally less intensive therapy and lower Hgb treatment targets [17, 18]. Following this change, high-quality longitudinal real-world data on the current impact of anemia on clinical outcomes are scarce. Furthermore, the exact risk of cardiovascular and other adverse outcomes in patients following progression to more severe anemia remains unknown.

The overall aim of our study was to identify major clinical consequences of anemia in dialysis dependent (DD) and non-dialysis dependent (NDD) patients with severe CKD. Specifically, we examined the association between anemia and time to first dialysis (in NDD patients) and major cardiovascular events (MACE), acute hospitalizations and all-cause death (in NDD and DD patients).

## Methods

We linked Danish population-based healthcare and administrative databases including all laboratory plasma-creatinine tests from primary and hospital care from The Laboratory Information Systems Database (LABKA) [19], all hospital contacts and diagnoses from The Danish National Patient Registry (DNPR), and all drug prescriptions from The Aarhus University Prescription Database [20, 21] for the entire population in Northern Denmark from 2000 through 2016 where residence, migration and vital status was obtained from The Civil Registration System (CRS). The cumulative source population comprised of ~ 2.2 million persons from which we included a study population with prevalent severe CKD at study start on Jan 1, 2009 (identified from 2000 to 2008), or with incident severe CKD between 2009 and 2016.

### Study population

Patients with severe CKD were defined as individuals with two plasma-creatinine tests at least 3 months (90 days) apart showing an estimated glomerular filtration rate (eGFR) < 30 mL/min/1.73 m^2^ in the period 2000–2016. eGFR was computed by the simplified Modification of Diet in Renal Disease (MDRD) equation based on creatinine measurements, taking sex and age and into account [22].

During 2009–2016, we assigned CKD patients (who had either prevalent severe CKD on Jan 1, 2009 or incident severe CKD between 2009 and 2016) to different anemia grade cohorts.

We excluded patients with any of the following at any time prior to the index date: any cancer (except non-melanoma skin cancer), hereditary hematologic disease, chronic inflammatory disease, gastrointestinal bleeding, or organ transplants.

### Anemia

**Fig. 1:**
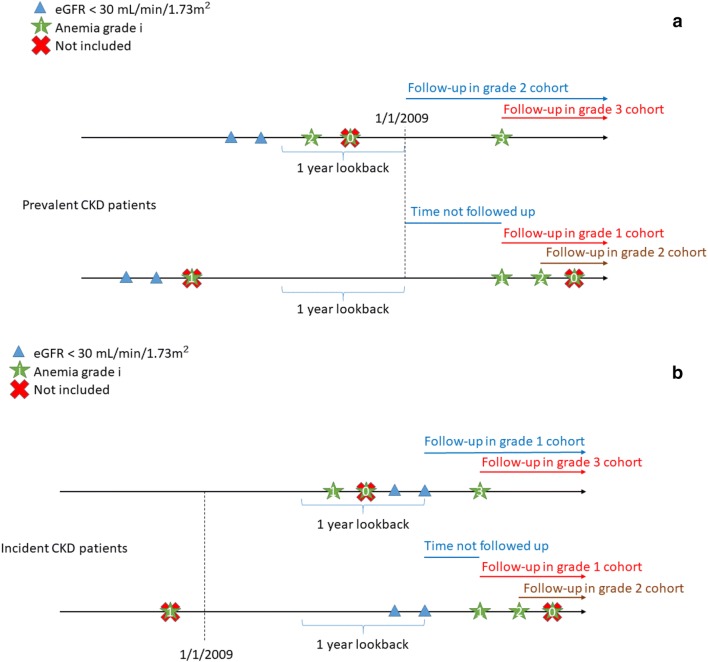
Overview of the inclusion based on eGFR and anemia grade of prevalent (**a**) and incident (**b**) severe CKD patients for follow up showing examples of how included patients may move to a more severe anemia grade cohort

Anemia grades were classified as no anemia (Hgb level of ≥ 12/≥ 13 g/dl in women/men); grade 1 (Hgb level of 10 to < 12/< 13 g/dl in women/men); grade 2 (Hgb level of 8 to < 10 g/dl) and grade 3+ (Hgb level of < 8 g/dl) [23]. For prevalent severe CKD patients, anemia grade was determined based on the lowest Hgb recording during a 1 year lookback period from Jan 1st 2009. If Hgb was not measured in this period, the follow up started following the first recorded Hgb measurement after Jan 1st 2009. For incident severe CKD patients, follow up began on the date of the second measurement showing an eGFR < 30 mL/min/1.73 m^2^ after Jan 1st 2009 if Hgb measurements were recorded within 1 year before that date. For these patients, anemia grade was determined based on the lowest Hgb recording during that year. If no prior Hgb measurements were available, follow up started following the first available Hgb measurement after the patient’s second eGFR measurement below 30 mL/min/1.73 m^2^. A schematic presentation of inclusion of prevalent severe CKD patients is shown in Fig. 1a and similarly for incident severe CKD patients in Fig. 1b. Patients could be included in more than one anemia grade cohort if they “moved up” in anemia grade; i.e., one person was allowed to contribute risk time in consecutively more severe anemia grade cohorts. Since our aim was to predict the subsequent outcome risk for a given CKD patient from the date of first reaching a specific anemia grade, patients were not censored from the cohort at time following inclusion into a more severe anemia grade cohort and were not re-entered into a lower anemia grade cohort once they were included in a more severe grade cohort irrespective of the results of subsequent Hgb measurements.

### Outcomes

Study outcomes were: (1) incident dialysis (among NDD patients only) defined as a first ever record of dialysis (acute or chronic) during follow-up; (2) all-cause acute hospitalization, defined as an acute inpatient record in DNPR during follow-up; (3) all-cause death, defined as death before the end of follow-up; (4) MACE (composite of tenth revision of the International Classification of Diseases and Related Health Problems (ICD-10) codes for myocardial infarction [I21–I23], unstable angina pectoris [I200], stroke [I61, I63 and I64], or heart failure [I50]); and (5) fatal and non-fatal cardiovascular events (CVE) separately (composite and individual). Patients who were already hospitalized at the index date were not considered at risk of an acute hospitalization and thus excluded from this analyses.

### Data analysis

For the incident severe CKD patients, we generated cumulative incidence function curves for incident dialysis among NDD patients and for CVE and all-cause death stratified by dialysis status at time of entry in each anemia grade cohort.

For both prevalent and incident severe CKD patients, we computed incidence rates per 100 person years and estimated adjusted hazard ratios (HRs) using Cox regression. To account for the dependency between observations, we used the robust sandwich estimator to compute 95% CIs [24]. HRs were simultaneously adjusted for the following covariates assessed at the start of follow-up in each anemia grade cohort: age, gender, marital status, severe CKD duration (time since 2nd eGFR < 30 mL/min/1.73 m^2^), last measured eGFR level, Charlson Comorbidity Index [25] (based on complete hospital contact history, excluding CVE and renal disease categories), alcohol dependency related disorders, previous history of CVE and number of acute hospitalizations 1 year before anemia.

Since the proportional hazard assumption was not fulfilled for incident dialysis, angina and acute hospitalization in the NDD population and for all outcomes in the DD population, supplementary analyses were performed dividing the analyses into 0–2 years and 2–8 years of follow-up.

Since the average age of the included study population was relatively high we performed a stratified analyses by ages < 65 and 65 + years, and as diabetes is associated with increased baseline risk of the included outcome events we also performed stratified analyses by diabetes status.

## Results

In total, we included data from 16,972 unique patients with severe CKD, who contributed to one or more of the anemia grade cohorts over time, resulting in a total of 28,510 patient profiles across all anemia cohorts. These included 24,916 (87%) NDD and 3594 (13%) DD patients, with different anemia grades. Characteristics are shown for all patients in Table 1, for NDD patient profiles in Supplementary Table 1, and for DD patient profiles in Supplementary Table 2.

**Table 1:** Characteristics of the 28,510 severe CKD patient profiles included in the study by first observation of anemia grade 0, 1, 2 or 3+ following severe CKD^a^

	No anemia = grade 0 (Hgb ≥ 13/≥ 12 g/dL)^b^	Anemia grade 1 (Hgb 10 to < 13/< 12 g/dL)^b^	Anemia grade 2 (Hgb 8 to < 10 g/dL)	Anemia grade 3+ (Hgb < 8 g/dL)	Total
Overall, N	4105	10,033	9632	4740	28,510
Hgb g/dl, median (IQR)	13.5 (12.9–14.5)	11.8 (11.3–12.1)	9.7 (9.3–10.0)	7.6 (7.3–7.9)	10.0 (9.3–11.9)
Age, median (IQR)	76 (13.8)	77 (13.7)	77 (13.8)	74 (14.5)	76 (13.9)
Male sex, N (%)	1485 (36.2)	4700 (46.8)	4380 (45.5)	2251 (47.5)	12,816 (45.0)
Marrital status					
Divorced, N (%)	431 (10.5)	1033 (10.3)	1038 (10.8)	601 (12.7)	3103 (10.9)
Married, N (%)	1566 (38.1)	3954 (39.4)	3610 (37.5)	1833 (38.7)	10,963 (38.5)
Unmarried, N (%)	406 (9.9)	1088 (10.8)	1118 (11.6)	676 (14.3)	3288 (11.5)
Widowed, N (%)	1702 (41.5)	3958 (39.4)	3866 (40.1)	1630 (34.4)	11,156 (39.1)
Severe CKD duration (months), median (IQR)	0 (0–26)	0 (0–29)	9 (0–42)	15 (0–50)	4 (0–37)
Dialysis dependent, N (%)	239 (5.8)	725 (7.2)	1326 (13.8)	1304 (27.5)	3594 (12.6)
Latest eGFR ml/min/1.73 m^2^ level before cohort entry					
60 + , N (%)	154 (3.8)	222 (2.2)	253 (2.6)	167 (3.5)	796 (2.8)
45 ≤ 60, N (%)	213 (5.2)	433 (4.3)	534 (5.5)	256 (5.4)	1436 (5.0)
30 ≤ 45, N (%)	670 (16.3)	1656 (16.5)	1673 (17.4)	754 (15.9)	4753 (16.7)
15 ≤ 30, N (%)	2941 (71.6)	6831 (68.1)	5209 (54.1)	2228 (47.0)	17,209 (60.4)
< 15, N (%)	127 (3.1)	891 (8.9)	1963 (20.4)	1335 (28.2)	4316 (15.1)
eGFR, Median (IQR)	28.2 (25.1–30.1)	27.1 (22.3–29.8)	25.3 (17.0–30.3)	23.0 (13.6–30.0)	26.5 (19.8–29.9)
Charlson comorbidity index^c^					
0, N (%)	1935 (47.1)	4023 (40.1)	3325 (34.5)	1380 (29.1)	10,663 (37.4)
1–2, N (%)	1794 (43.7)	4674 (46.6)	4465 (46.4)	2250 (47.5)	13,183 (46.2)
3+ , N (%)	376 (9.2)	1336 (13.3)	1842 (19.1)	1110 (23.4)	4664 (16.4)
Diabetes, N (%)	1163 (28.3)	3349 (33.4)	3451 (35.8)	1843 (38.9)	9806 (34.4)
History of heart failure, N (%)	826 (20.1)	2310 (23.0)	2378 (24.7)	1138 (24.0)	6652 (23.3)
History of CVD, N (%)	1816 (44.2)	4905 (48.9)	4985 (51.8)	2419 (51.0)	14,125 (49.5)
Number of acute hospitalizations one year back, median (IQR)	0 (0–1)	0 (0–1)	1 (1, 2)	2 (1–3)	1 (0–2)
Alcoholism related disorders, N (%)	290 (7.1)	787 (7.8)	924 (9.6)	612 (12.9)	2613 (9.2)
Antihypertensive/antilipid-/antiplatelet therapy, N (%)	3868 (94.2)	9560 (95.3)	9197 (95.5)	4525 (95.5)	27,150 (95.2)

^a^Severe CKD was defined based on the 2nd of two eGFR calculations at least 3 months apart showing a eGFR < 30. At the date of first observation in each anemia grade, eGFR may be higher than 30 as shown in the table

^b^Hgb ≥ 13 g/dL for men and ≥ 12 g/dL for women

^c^Charlson comorbidity score was calculated based on complete hospital contact history, excluding cardiovascular disease and renal disease categories

Overall we identified 4105 (14%) patient profiles with no anemia, 10,033 (35%) with anemia grade 1, 9631 (34%) with anemia grade 2 and 4740 (17%) with anemia grade 3+.

The duration of severe CKD, the number of acute hospitalizations (within the last year), the proportion of patients on dialysis, with low eGFR (< 15 ml/min/1.73 m^2^) and with a high comorbidity score all increased with increasing anemia grade (Table 1).

The incidence rates of incident dialysis among NDD patients and of acute hospitalization, all-cause deaths and MACE among all patients (Table 2) increased markedly with increasing anemia grades both when analyzing crude incidence rates per 100 person years (IR) and hazard ratios for clinical outcomes by anemia grade, and when these were adjusted for baseline differences in potential confounders (Table 2). The adjusted estimates were attenuated compared to the crude estimates. Among the NDD patients, the IR for newly initiated dialysis at anemia grade 3+ was 8.6 per 100, 95% CI 7.8–9.5 and the adjusted HR was markedly elevated (HR 1.91, 95% CI 1.61–2.26) at anemia grade 3+ when compared to no anemia. Anemia was strongly associated with acute hospitalization for any cause (IR at anemia grade 3 + 103.9, 95% CI 96.4–111.8 and HR 1.74, 95% CI 1.57–1.93) and with all-cause death (IR at anemia grade 3+ 39.4, 95% CI 37.8–41.1 and HR 1.82, 95% CI 1.70–1.94). The HR for MACE at anemia grade 3+ was only modestly elevated, although statistically significant (IR at anemia grade 3+ 15.4, 95% CI 14.3–16.6 and HR 1.14, 95% CI 1.02–1.26). Among the individual MACE endpoints, heart failure revealed the strongest association with anemia (IR at anemia grade 3+ 10.7, 95% CI 9.8–11.6 and HR 1.24, 95% CI 1.09–1.41).

**Table 2:** Crude Incidence rate as well as crude and adjusted^a^ hazard ratios for incident dialysis, any and specific cardiovascular events, acute hospitalization and all-cause death associated with different anemia grades among non-dialysis dependent patients with severe CKD

	N	No. of events	Follow-up time, years	Crude incidence rate per 100 person-years (95% CI)	Crude hazard ratio (95% CI)	Adjusted hazard ratio (95% CI)^a^
Incident dialysis						
No anemia	3866	283	12,380	2.3 (2.0–2.6)	(ref)	(ref)
Anemia grade 1	9308	977	24,066	4.1 (3.8–4.3)	1.71 (1.55–1.90)	1.27 (1.14–1.42)
Anemia grade 2	8306	1060	15,618	6.8 (6.4–7.2)	2.70 (2.41–3.03)	1.69 (1.49–1.93)
Anemia grade 3+	3436	435	5043	8.6 (7.8–9.5)	3.26 (2.83–3.76)	1.91 (1.61–2.26)
Acute hospitalization						
No anemia	3111	2187	6203	35.3 (33.8–36.8)	(ref)	(ref)
Anemia grade 1	6370	4609	9937	46.4 (45.1–47.7)	1.24 (1.19–1.30)	1.12 (1.07–1.17)
Anemia grade 2	3709	2795	3816	73.2 (70.5–76.0)	1.79 (1.69–1.89)	1.41 (1.33–1.50)
Anemia grade 3+	943	713	686	103.9 (96.4–111.8)	2.41 (2.19–2.65)	1.74 (1.57–1.93)
All-cause death						
No anemia	3866	1921	12,939	14.8 (14.2–15.5)	(ref)	(ref)
Anemia grade 1	9308	5210	26,326	19.8 (19.3–20.3)	1.29 (1.24–1.35)	1.15 (1.10–1.19)
Anemia grade 2	8306	5331	17,760	30.0 (29.2–30.8)	1.86 (1.78–1.95)	1.47 (1.40–1.55)
Anemia grade 3+	3436	2275	5770	39.4 (37.8–41.1)	2.32 (2.19–2.47)	1.82 (1.70–1.94)
Cardiovascular events						
No anemia	3866	898	11,541	7.8 (7.3–8.3)	(ref)	(ref)
Anemia grade 1	9308	2304	22,867	10.1 (9.7–10.5)	1.23 (1.15–1.31)	1.07 (1.00–1.14)
Anemia grade 2	8306	1943	15,199	12.8 (12.2–13.4)	1.44 (1.34–1.55)	1.10 (1.02–1.19)
Anemia grade 3+	3436	749	4855	15.4 (14.3–16.6)	1.62 (1.47–1.78)	1.14 (1.02–1.26)
Myocardial infarction						
No anemia	3866	204	12,620	1.6 (1.4–1.9)	(ref)	(ref)
Anemia grade 1	9308	534	25,523	2.1 (1.9–2.3)	1.25 (1.09–1.43)	1.10 (0.96–1.26)
Anemia grade 2	8306	451	17,146	2.6 (2.4–2.9)	1.48 (1.27–1.73)	1.20 (1.01–1.42)
Anemia grade 3+	3436	148	5593	2.6 (2.2–3.1)	1.41 (1.15–1.75)	1.11 (0.88–1.39)
Stroke						
No anemia	3866	255	12,602	2.0 (1.8–2.3)	(ref)	(ref)
Anemia grade 1	9308	550	25,581	2.2 (2.0–2.3)	1.03 (0.92–1.16)	0.95 (0.84–1.07)
Anemia grade 2	8306	440	17,214	2.6 (2.3–2.8)	1.17 (1.01–1.35)	0.98 (0.84–1.15)
Anemia grade 3+	3436	157	5574	2.8 (2.4–3.3)	1.23 (1.01–1.49)	1.01 (0.81–1.25)
Heart failure						
No anemia	3866	561	12,109	4.6 (4.3–5.0)	(ref)	(ref)
Anemia grade 1	9308	1543	24,094	6.4 (6.1–6.7)	1.31 (1.21–1.41)	1.11 (1.03–1.20)
Anemia grade 2	8306	1337	16,067	8.3 (7.9–8.8)	1.55 (1.42–1.70)	1.15 (1.04–1.27)
Anemia grade 3+	3436	547	5125	10.7 (9.8–11.6)	1.84 (1.64–2.06)	1.24 (1.09–1.41)
Unstable angina pectoris						
No anemia	3866	40	12,826	0.3 (0.2–0.4)	(ref)	(ref)
Anemia grade 1	9308	84	26,136	0.3 (0.3–0.4)	1.00 (0.75–1.35)	0.88 (0.65–1.20)
Anemia grade 2	8306	55	17,657	0.3 (0.2–0.4)	0.93 (0.63–1.37)	0.74 (0.48–1.15)
Anemia grade 3+	3436	20	5731	0.3 (0.2–0.5)	1.00 (0.58–1.70)	0.71 (0.38–1.32)

^a^Adjusted for age, gender, marital status, history of CVD, alcohol dependency, other comorbidities, recent acute hospitalizations, eGFR level and CKD duration

For the DD population (Table 3), similar risk estimates as in the NDD population were observed for acute hospitalization (IR 121.9, 95% CI 109.8–135.0 and HR 1.51, 95% CI 1.20–1.90) all-cause death (IR 23.4, 95% CI 21.6–25.0 and HR 1.91, 95% CI 1.50–2.43) and MACE (IR 10.4, 95% CI 9.3–11.7 and HR 1.16, 95% CI 0.84–1.61) although the estimates were less precise, probably due to lower number of subjects included in the analyses.

**Table 3:** Crude Incidence rate as well as crude and adjusted^a^ and hazard ratios for any and specific cardiovascular events, acute hospitalization and all-cause death associated with different anemia grades among dialysis dependent patients

	N	No. of events	Follow-up time, years	Crude incidence rate per 100 person-years (95% CI)	Crude hazard ratio (95% CI)	Adjusted hazard ratio (95% CI)^a^
Acute hospitalization						
No anemia	199	155	551	28.1 (23.9–32.9)	(ref)	(ref)
Anemia grade 1	584	517	924	56.0 (51.3–61.0)	1.68 (1.44–1.95)	1.09 (0.92–1.29)
Anemia grade 2	720	662	735	90.0 (83.3–97.2)	2.37 (2.02–2.78)	1.21 (0.99–1.47)
Anemia grade 3+	434	371	304	121.9 (109.8–135.0)	2.99 (2.47–3.61)	1.51 (1.20–1.90)
All-cause death						
No anemia	239	86	1300	6.6 (5.3–8.2)	(ref)	(ref)
Anemia grade 1	725	390	3293	11.8 (10.7–13.1)	1.73 (1.42–2.11)	1.29 (1.05–1.58)
Anemia grade 2	1326	770	4476	17.2 (16.0–18.5)	2.36 (1.92–2.91)	1.52 (1.22–1.91)
Anemia grade 3+	1304	712	3060	23.3 (21.6–25.0)	2.95 (2.37–3.68)	1.91 (1.50–2.43)
Cardiovascular events						
No anemia	239	49	1217	4.0 (3.0–5.3)	(ref)	(ref)
Anemia grade 1	725	183	2928	6.2 (5.4–7.2)	1.43 (1.09–1.88)	1.03 (0.78–1.37)
Anemia grade 2	1326	361	3873	9.3 (8.4–10.3)	1.92 (1.44–2.54)	1.16 (0.86–1.57)
Anemia grade 3+	1304	280	2682	10.4 (9.3–11.7)	1.92 (1.43–2.59)	1.16 (0.84–1.61)
Myocardial infarction						
No anemia	239	11	1283	0.9 (0.4–1.5)	(ref)	(ref)
Anemia grade 1	725	64	3196	2.0 (1.5–2.6)	2.22 (1.24–3.96)	1.15 (0.62–2.16)
Anemia grade 2	1326	102	4329	2.4 (1.9–2.9)	2.40 (1.30–4.41)	1.03 (0.52–2.03)
Anemia grade 3+	1304	67	2970	2.3 (1.7–2.9)	2.06 (1.08–3.95)	0.91 (0.44–1.90)
Stroke						
No anemia	239	19	1264	1.5 (0.9–2.3)	(ref)	(ref)
Anemia grade 1	725	63	3153	2.0 (1.5–2.6)	1.26 (0.82–1.95)	0.90 (0.59–1.37)
Anemia grade 2	1326	122	4261	2.9 (2.4–3.4)	1.68 (1.08–2.61)	1.06 (0.68–1.65)
Anemia grade 3+	1304	88	2935	3.0 (2.4–3.7)	1.62 (1.01–2.59)	1.04 (0.63–1.71)
Heart failure						
No anemia	239	27	1262	2.1 (1.4–3.1)	(ref)	(ref)
Anemia grade 1	725	98	3118	3.1 (2.6–3.8)	1.37 (0.95–1.97)	1.17 (0.80–1.71)
Anemia grade 2	1326	208	4175	5.0 (4.3–5.7)	1.97 (1.34–2.89)	1.45 (0.96–2.19)
Anemia grade 3+	1304	167	2879	5.8 (5.0–6.7)	2.05 (1.37–3.07)	1.53 (0.98–2.40)
Unstable angina pectoris						
No anemia^b^	239	1	1299	0.1 (0.0–0.4)	–	–
Anemia grade 1	725	16	3242	0.5 (0.3–0.8)	(ref)	(ref)
Anemia grade 2	1326	27	4396	0.6 (0.4–0.9)	1.11 (0.73–1.68)	0.87 (0.55–1.38)
Anemia grade 3+	1304	17	3013	0.6 (0.3–0.9)	0.91 (0.50–1.66)	0.69 (0.35–1.36)

^a^Adjusted for age, gender, marital status, history of CVD, alcohol dependency, other comorbidities, recent acute hospitalizations, eGFR level and CKD duration

^b^Due to low number of events in the no anemia group the Anemia grade 1 group is used as reference

Fatal MACE were more strongly associated with anemia (HR 1.42, 95% CI 1.19–1.68) than non-fatal events (Supplementary Tables 3 and 4). In particular, fatal events of stroke, myocardial infarction and unstable angina were associated with anemia, while similar non-fatal events revealed HRs close to one. Both fatal and non-fatal heart failure events were clearly associated with anemia in both the NDD and the DD patients.

In supplementary analyses, outcomes were evaluated based on follow up time dividing this into 0–2 years and 2–8 years. Among the NDD patients, the HRs for incident dialysis, acute hospitalization and all-cause death associated with anemia grade 3+ compared to no anemia were markedly higher during the first 2 years of follow up when compared to 2–8 years (Supplementary Table 5). Similarly, in DD patients higher HRs for acute hospitalization and all-cause death associated with anemia were observed during the first 2 years when compared to 2–8 years (Supplementary Table 6).

Stratification by age < 65 years and 65+ years and by presence or absence of diabetes revealed similar results to the comparator, indicating that these were not key effect modifiers (data not shown). NDD patients with a known hospital history of heart failure at baseline had lower HRs than those without previously known heart failure for death (HR 1.55, 95% CI 1.38–1.74 vs HR 1.97, 95% CI 1.82–2.13), incident heart failure hospitalization (HR 1.11, 95% CI 0.93–1.32 vs HR 1.55, 95% CI 1.28–1.88) and MACE (HR 1.04, 95% CI 0.89–1.23 vs HR 1.29, 95% CI 1.12–1.48). For the DD population similar but less precise estimates were obtained (data not shown).

**Fig. 2:**
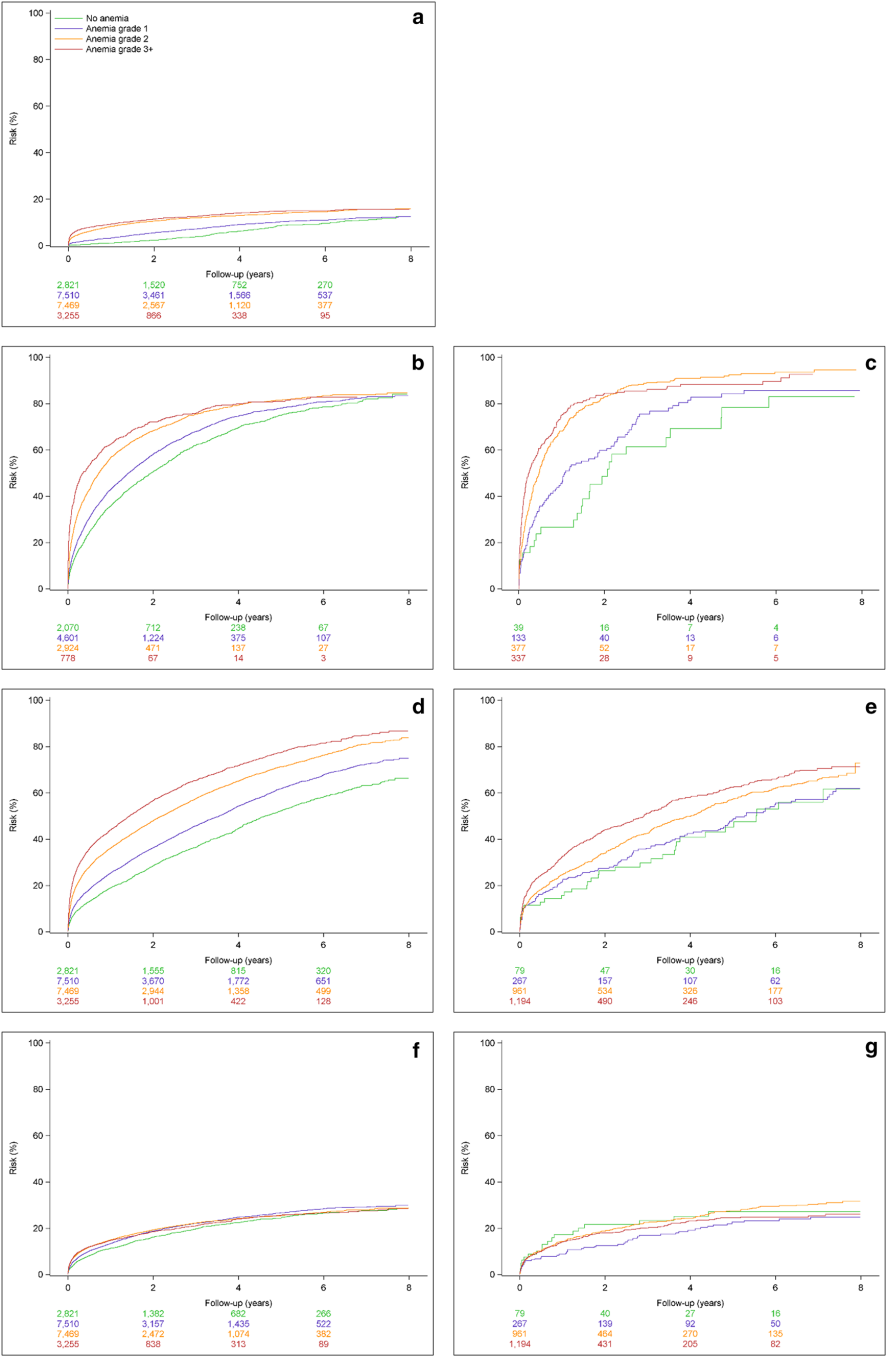
Cumulative incidence curves for patients with incident severe CKD. **a** incident dialysis for non-dialysis dependent patients; **b** acute hospitalization for non-dialysis dependent patients; **c** acute hospitalization for dialysis dependent patients; **d** all-cause mortality for non-dialysis dependent patients; **e** all-cause mortality for dialysis dependent patients; **f** cardiovascular events for non-dialysis dependent patients; **g** cardiovascular events for dialysis dependent patients

Cumulative incidence function curves for study outcomes in NDD and DD patients are shown in Fig. 2a–g. The risk of dialysis initiation in NDD patients as well as the risks for acute hospitalization and all-cause death among both NDD and DD increases successively with increasing anemia grades. The risk of MACE increased modestly with higher anemia grades among NDD patients, while no clear association was observed among DD patients (Fig. 2a–e).

## Discussion

In the current setting of less frequent treatment of NDD anemia and treatment to lower Hgb targets among patients with severe CKD and anemia, the present study provides contemporary data showing that increasing grades of anemia in severe CKD patients are associated with increased absolute and relative risks of a number of adverse health outcomes, including dialysis initiation, MACE, acute hospitalization and all-cause death. In general, similar results were seen among DD and NDD patients.

Compared with previous cross-sectional studies of anemia prevalence in CKD, we observed higher proportions of severe CKD patients experiencing grade 2 or 3 anemia at any given point of time, likely due to the longitudinal nature of our study enabling us to identify incident anemia events. Our results corroborate earlier findings on the association between anemia and clinical outcomes in smaller and selected populations from the United States or Japan [5, 11–13].

The observed and markedly increased HR of incident dialysis, acute hospitalization, all-cause death and MACE during the first 2 years after anemia may indicate that CKD four to five patients surviving the first 2 years after anemia have an only slightly elevated, subsequent risk of adverse events when compared to non-anemic patients. This interpretation, however, is limited by the fact that only survivors were followed in this study. In addition, in this study anemia grade was defined by the lowest Hgb level observed during 1 year before inclusion. This Hgb level may not have been constant during the follow-up period. Since our analyses did not differentiate between patients with sustained and chronic anemia and patients with acute anemia or anemia that was corrected during follow up, our risk estimates may have underestimated the effects of chronic anemia on long term adverse outcomes. Future studies should look into the causes and mechanisms through which increased anemia associates with acute hospitalizations and death, other than CVE.

This study was a population-based study using extensive registry health data. This is a major strength of the study since the universal health care system in Denmark implies that all individuals with contact to the hospital system are included in the registers thereby providing an unselected real-world study population. Also, compared to previous studies we were able to include a large study population of 16,930 patients with severe CKD, with ascertainment of progressing anemia over time. We could follow these patients longitudinally for up to 8 years after anemia and adjust our outcome estimates for a large set of relevant confounders. In general, the registration of exposures and outcomes in the included databases is accurate and the validity of registration of major diagnoses including cardiovascular diseases in the Danish National Patient Registry is high [26, 27].

Several limitations also apply to the study. The inclusion of severe CKD patients relied on routine plasma-creatinine measurements and some misclassification of CKD patients may occur based on missing or infrequent plasma creatinine measurements to determine CKD stage. Although the definition of CKD was based on registered eGFR measurements and the diagnosis was not verified by a nephrologist, we do not expect this to be associated to major misclassification of CKD, since we used the common definition of CKD as specified above. The estimation of GFR was based on the MDRD equation, which was the predominant equation used in clinical practice throughout the period of data collection. Currently, the CKD-EPI equation is more often used. The divergence between the equations occurs mainly at eGFR values > 30 mL/min/1.73 m^2^ [28], and thus, this most likely has limited impact on the results of the present study.

Sicker patients with more severe CKD may have had a higher likelihood of any anemia being detected due to higher frequency of Hgb testing, leading to potential overestimation of anemia and possibly also its clinical consequences. However, our patients all had severe CKD and are all likely to receive regular blood test monitoring including Hgb in a universally covering healthcare system. We did not have complete treatment data for ESAs, iron, and transfusions recorded in our prescription registries. Therefore, the effects of anemia alone from the potential adverse effects of anemia treatment could not be differentiated in our study. It is likely that patients with severe anemia and late stage CKD did receive ESAs, IV iron or RBC transfusions occasionally, as is suggested by international guidelines [29, 30]. Overall, our results suggest that higher Hgb levels are associated with better outcomes; however, the potential positive or negative effects of anemia treatment cannot be assessed from this study.

Finally, we did not account for differences in blood pressure and proteinuria, and we cannot exclude unmeasured confounding from e.g. smoking, socioeconomic status or lifestyle factors on which did not have reliable data, or from other, unknown confounding factors.

## Conclusions

The presence and increasing severity of anemia were associated with a substantially increased risk for progression to dialysis in non-dialysis patients with CKD stage 4–5, and with an increased risk for acute hospitalization and all-cause death both in non-dialysis and dialysis patients. The presence and increasing severity of anemia was also associated with a moderately elevated risk of MACE which was more pronounced during the first 2 years after entering a lower anemia grade and with particularly increased risks of heart failure and fatal CVE. In the current setting of generally less intensive anemia therapy, the present large study emphasizes the need for awareness of the potential risk of adverse clinical events in patients with CKD and anemia, and for further exploration of the interaction between anemia and progressive disease in CKD.

## Electronic supplementary material

Below is the link to the electronic supplementary material.

Supplementary material 1 (DOCX 52 kb)
