# Supplementary material for: Anemia and clinical outcomes in patients with non-dialysis dependent or dialysis dependent severe chronic kidney disease: a Danish population-based study
Source: J Nephrol. 2019 Oct 5;33(1):147–56. doi: 10.1007/s40620-019-00652-9 (PMC7007417; doi:10.1007/s40620-019-00652-9)
Supplement: Supplementary file 1 — Supplementary material 1 (DOCX 52 kb) [file 40620_2019_652_MOESM1_ESM.docx]

# Supplementary Material

# Anemia and Clinical Outcomes in Patients with Non-dialysis Dependent or Dialysis Dependent Severe Chronic Kidney Disease: A Danish Population-based Study

**Table of contents**

**Page 2: Supplementary Table 1** Characteristics of the 24,916 non-dialysis dependent severe CKD patient profiles included in the study by first observation of anemia grade 0, 1, 2 or 3+ following severe CKD

**Page 3: Supplementary Table 2** Characteristics of the 3,594 non-dialysis dependent severe CKD patient profiles included in the study by first observation of anemia grade 0, 1, 2 or 3+ following severe CKD

**Page 4: Supplementary Table 3** Crude Incidence rate as well as crude and adjusted hazard ratios for any and specific fatal and nonfatal cardiovascular events associated with different anemia grades among non-dialysis dependent severe CKD patients

**Page 6: Supplementary Table 4** Crude Incidence rate as well as crude and adjusted hazard ratios for any and specific fatal and nonfatal cardiovascular events associated with different anemia grades among dialysis dependent patients

**Page 8: Supplementary Table 5** Crude and adjusted hazard ratios for incident dialysis, all cause death, any and specific cardiovascular events stratified by fatal and non-fatal events associated with different anemia grades among non-dialysis dependent severe CKD patients split by follow up time (0-2 years and 2-8 years)

**Page 12: Supplementary Table 6** Crude and adjusted hazard ratios for all-cause death, any and specific cardiovascular events associated with different anemia grades among dialysis dependent patients stratified by follow up time (0-2 years and 2-8 years)

**Supplementary Table 1** Characteristics of the 24,916 non-dialysis dependent severe CKD patient profiles included in the study by first observation of anemia grade 0, 1, 2 or 3+ following severe CKD^a^

|  | **No anemia = grade 0 (Hgb≥13/≥12g/dL)^b^** | **Anemia grade 1 (Hgb 10 to <13/<12 g/dL)^b^** | **Anemia grade 2 (Hgb 8 to <10 g/dL)** | **Anemia grade 3+ (Hgb <8 g/dL)** | **Total** |
| --- | --- | --- | --- | --- | --- |
| Overall, N | 3,866 | 9,308 | 8,306 | 3,436 | 24,916 |
| Hgb g/dl, Median (IQR) | 13.5 (12.9 - 14.3) | 11.8 (11.3 - 12.1) | 9.7 (9.3 - 10.0) | 7.6 (7.3 - 7.9) | 10.6 (9.5 - 12.1) |
| Age, Median (IQR) | 79 (71 - 86) | 80 (72 - 87) | 81 (73 - 87) | 80 (71 - 86) | 80 (72 - 87) |
| Male sex, N (%) | 1,348 (34.9) | 4,239 (45.5) | 3,551 (42.8) | 1,461 (42.5) | 10,599 (42.5) |
| Marital status |  |  |  |  |  |
| Divorsed, N (%) | 406 (10.5) | 939 (10.1) | 850 (10.2) | 404 (11.8) | 2,599 (10.4) |
| Married, N (%) | 1,427 (36.9) | 3,583 (38.5) | 2,932 (35.3) | 1,207 (35.1) | 9,149 (36.7) |
| Unmarried, N (%) | 368 (9.5) | 949 (10.2) | 863 (10.4) | 404 (11.8) | 2,584 (10.4) |
| Widower, N (%) | 1,665 (43.1) | 3,837 (41.2) | 3,661 (44.1) | 1,421 (41.4) | 10,584 (42.5) |
| Severe CKD duration (months), Median (IQR) | 0 (0 - 21) | 0 (0 - 23) | 6 (0 - 34) | 9 (0 - 38) | 1 (0 - 29) |
| Latest eGFR (ml/min/1.73m^2^) level before cohort entry |  |  |  |  |  |
| 60+, N (%) | 101 (2.6) | 188 (2.0) | 222 (2.7) | 136 (4.0) | 647 (2.6) |
| 45-<60, N (%) | 174 (4.5) | 372 (4.0) | 484 (5.8) | 218 (6.3) | 1,248 (5.0) |
| 30-<45, N (%) | 624 (16.1) | 1,542 (16.6) | 1,563 (18.8) | 659 (19.2) | 4,388 (17.6) |
| 15-<30, N (%) | 2,863 (74.1) | 6,647 (71.4) | 4,852 (58.4) | 1,844 (53.7) | 16,206 (65.0) |
| <15, N (%) | 104 (2.7) | 559 (6.0) | 1,185 (14.3) | 579 (16.9) | 2,427 (9.7) |
| eGFR, Median (IQR) | 28.1 (25.1 - 29.9) | 27.2 (22.9 - 29.8) | 26.2 (19.4 - 31.1) | 25.8 (18.3 - 32.4) | 27.1 (21.7 - 30.1) |
| Charlson comorbidity index^c^ |  |  |  |  |  |
| 0, N (%) | 1,830 (47.3) | 3,771 (40.5) | 2,893 (34.8) | 995 (29.0) | 9,489 (38.1) |
| 1-2, N (%) | 1,687 (43.6) | 4,343 (46.7) | 3,869 (46.6) | 1,670 (48.6) | 11,569 (46.4) |
| 3+, N (%) | 349 (9.0) | 1,194 (12.8) | 1,544 (18.6) | 771 (22.4) | 3,858 (15.5) |
| Diabetes, N (%) | 1,096 (28.3) | 3,098 (33.3) | 2,970 (35.8) | 1,266 (36.8) | 8,430 (33.8) |
| History of heart failure, N (%) | 792 (20.5) | 2,198 (23.6) | 2,111 (25.4) | 876 (25.5) | 5,977 (24.0) |
| History of CVD, N (%) | 1,725 (44.6) | 4,575 (49.2) | 4,339 (52.2) | 1,809 (52.6) | 12,448 (50.0) |
| Number of acute hospitalizations one year back, Median (IQR) | 0 (0 - 1) | 0 (0 - 1) | 1 (0 - 2) | 2 (1 - 3) | 1 (0 - 2) |
| Alcoholism related disorders, N (%) | 273 (7.1) | 723 (7.8) | 808 (9.7) | 452 (13.2) | 2,256 (9.1) |
| Antihypertensive/antilipid-/antiplatelet therapy, N (%) | 3,651 (94.4) | 8,885 (95.5) | 7,937 (95.6) | 3,268 (95.1) | 23,741 (95.3) |

^a^ Severe CKD was defined based on the 2^nd^ of two eGFR calculations at least 3 months apart showing a eGFR<30. At the date of first observation in each anemia grade, eGFR may be higher than 30 as shown in the table.
^b^ Hgb≥13 g/dL for men and ≥12g/dL for women.
^c^Charlson comorbidity score was calculated based on complete hospital contact history, excluding cardiovascular disease and renal disease categories.

**Supplementary Table 2** Characteristics of the 3,594 non dialysis dependent severe CKD patient profiles included in the study by first observation of anemia grade 0, 1, 2 or 3+ following severe CKD^a^

|  | **No anemia = grade 0 (Hgb≥13/≥12g/dL)^b^** | **Anemia grade 1 (Hgb 10 to <13/<12 g/dL)^b^** | **Anemia grade 2 (Hgb 8 to <10 g/dL)** | **Anemia grade 3+ (Hgb <8 g/dL)** | **Total** |
| --- | --- | --- | --- | --- | --- |
| Overall, N | 239 | 725 | 1,326 | 1,304 | 3,594 |
| Hgb g/dl, Median (IQR) | 14.2 (13.4 - 15.0) | 11.9 (11.4 - 12.4) | 9.7 (9.3 - 10.0) | 7.7 (7.4 - 7.9) | 9.5 (7.9 - 10.8) |
| Age, Median (IQR) | 63 (50 - 74) | 66 (54 - 76) | 68 (57 - 76) | 67 (56 - 76) | 67 (56 - 76) |
| Male sex, N (%) | 137 (57.3) | 461 (63.6) | 829 (62.5) | 790 (60.6) | 2,217 (61.7) |
| Marital status |  |  |  |  |  |
| Divorsed, N (%) | 25 (10.5) | 94 (13.0) | 188 (14.2) | 197 (15.1) | 504 (14.0) |
| Married, N (%) | 139 (58.2) | 371 (51.2) | 678 (51.1) | 626 (48.0) | 1,814 (50.5) |
| Unmarried, N (%) | 38 (15.9) | 139 (19.2) | 255 (19.2) | 272 (20.9) | 704 (19.6) |
| Widower, N (%) | 37 (15.5) | 121 (16.7) | 205 (15.5) | 209 (16.0) | 572 (15.9) |
| Severe CKD duration (months), Median (IQR) | 47 (10 - 100) | 48 (25 - 95) | 46 (12 - 91) | 40 (2 - 84) | 45 (9 - 91) |
| Latest eGFR (ml/min/1.73m^2^) level before cohort entry |  |  |  |  |  |
| 60+, N (%) | 53 (22.2) | 34 (4.7) | 31 (2.3) | 31 (2.4) | 149 (4.1) |
| 45-<60, N (%) | 39 (16.3) | 61 (8.4) | 50 (3.8) | 38 (2.9) | 188 (5.2) |
| 30-<45, N (%) | 46 (19.2) | 114 (15.7) | 110 (8.3) | 95 (7.3) | 365 (10.2) |
| 15-<30, N (%) | 78 (32.6) | 184 (25.4) | 357 (26.9) | 384 (29.4) | 1,003 (27.9) |
| <15, N (%) | 23 (9.6) | 332 (45.8) | 778 (58.7) | 756 (58.0) | 1,889 (52.6) |
| eGFR, Median (IQR) | 37.8 (26.1 - 58.9) | 18.3 (7.5 - 33.7) | 11.3 (7.0 - 24.7) | 12.5 (7.6 - 22.2) | 13.6 (7.5 - 27.9) |
| Charlson comorbidity index^c^ |  |  |  |  |  |
| 0, N (%) | 105 (43.9) | 252 (34.8) | 432 (32.6) | 385 (29.5) | 1,174 (32.7) |
| 1-2, N (%) | 107 (44.8) | 331 (45.7) | 596 (44.9) | 580 (44.5) | 1,614 (44.9) |
| 3+, N (%) | 27 (11.3) | 142 (19.6) | 298 (22.5) | 339 (26.0) | 806 (22.4) |
| Diabetes, N (%) | 67 (28.0) | 251 (34.6) | 481 (36.3) | 577 (44.2) | 1,376 (38.3) |
| History of heart failure, N (%) | 34 (14.2) | 112 (15.4) | 267 (20.1) | 262 (20.1) | 675 (18.8) |
| History of CVD, N (%) | 91 (38.1) | 330 (45.5) | 646 (48.7) | 610 (46.8) | 1,677 (46.7) |
| Number of acute hospitalizations one year back, Median (IQR) | 0 (0 - 1) | 1 (0 - 2) | 2 (1 - 3) | 2 (1 - 4) | 2 (1 - 3) |
| Alcoholism related disorders, N (%) | 17 (7.1) | 64 (8.8) | 116 (8.7) | 160 (12.3) | 357 (9.9) |
| Antihypertensive/antilipid-/antiplatelet therapy, N (%) | 217 (90.8) | 675 (93.1) | 1,260 (95.0) | 1,257 (96.4) | 3,409 (94.9) |

^a^ Severe CKD was defined based on the 2^nd^ of two eGFR calculations at least 3 months apart showing a eGFR<30. At the date of first observation in each anemia grade, eGFR may be higher than 30 as shown in the table.
^b^ Hgb≥13 g/dL for men and ≥12g/dL for women.
^c^Charlson comorbidity score was calculated based on complete hospital contact history, excluding cardiovascular disease and renal disease categories.

**Supplementary Table 3** Crude Incidence rate as well as crude and adjusted^a^ hazard ratios for any and specific fatal and nonfatal cardiovascular events associated with different anemia grades among non-dialysis dependent severe CKD patients.

|  | **N** | **No. of events** | **Follow-up time, years** | **Crude incidence rate per 100 person-years (95% CI)** | **Crude hazard ratio (95% CI)** | **Adjusted hazard ratio (95% CI)^a^** |
| --- | --- | --- | --- | --- | --- | --- |
| **Incident dialysis** |  |  |  |  |  |  |
| No anemia | 3,866 | 283 | 12,380 | 2.3 (2.0 - 2.6) | (ref) | (ref) |
| Anemia grade 1 | 9,308 | 977 | 24,066 | 4.1 (3.8 - 4.3) | 1.71 (1.55 - 1.90) | 1.27 (1.14 - 1.42) |
| Anemia grade 2 | 8,306 | 1,060 | 15,618 | 6.8 (6.4 - 7.2) | 2.70 (2.41 - 3.03) | 1.69 (1.49 - 1.93) |
| Anemia grade 3+ | 3,436 | 435 | 5,043 | 8.6 (7.8 - 9.5) | 3.26 (2.83 - 3.76) | 1.91 (1.61 - 2.26) |
| **Fatal cardiovascular events (CVE)** |  |  |  |  |  |  |
| No anemia | 3,866 | 301 | 12,939 | 2.3 (2.1 - 2.6) | (ref) | (ref) |
| Anemia grade 1 | 9,308 | 842 | 26,326 | 3.2 (3.0 - 3.4) | 1.35 (1.21 - 1.50) | 1.13 (1.02 - 1.27) |
| Anemia grade 2 | 8,306 | 802 | 17,760 | 4.5 (4.2 - 4.8) | 1.84 (1.62 - 2.08) | 1.35 (1.18 - 1.54) |
| Anemia grade 3+ | 3,436 | 296 | 5,770 | 5.1 (4.6 - 5.7) | 2.02 (1.73 - 2.36) | 1.42 (1.19 - 1.68) |
| **Nonfatal CVE** |  |  |  |  |  |  |
| No anemia | 3,866 | 715 | 11,605 | 6.2 (5.7 - 6.6) | (ref) | (ref) |
| Anemia grade 1 | 9,308 | 1,793 | 23,029 | 7.8 (7.4 - 8.2) | 1.21 (1.13 - 1.30) | 1.05 (0.98 - 1.13) |
| Anemia grade 2 | 8,306 | 1,480 | 15,333 | 9.7 (9.2 - 10.2) | 1.41 (1.29 - 1.53) | 1.08 (0.99 - 1.18) |
| Anemia grade 3+ | 3,436 | 573 | 4,907 | 11.7 (10.7 - 12.7) | 1.61 (1.45 - 1.80) | 1.12 (0.99 - 1.26) |
| **Fatal myocardial infarction** |  |  |  |  |  |  |
| No anemia | 3,866 | 61 | 12,939 | 0.5 (0.4 - 0.6) | (ref) | (ref) |
| Anemia grade 1 | 9,308 | 173 | 26,326 | 0.7 (0.6 - 0.8) | 1.36 (1.07 - 1.74) | 1.21 (0.95 - 1.53) |
| Anemia grade 2 | 8,306 | 151 | 17,760 | 0.9 (0.7 - 1.0) | 1.70 (1.28 - 2.27) | 1.40 (1.03 - 1.89) |
| Anemia grade 3+ | 3,436 | 49 | 5,770 | 0.8 (0.6 - 1.1) | 1.64 (1.12 - 2.40) | 1.39 (0.92 - 2.08) |
| **Nonfatal myocardial infarction** |  |  |  |  |  |  |
| No anemia | 3,866 | 154 | 12,634 | 1.2 (1.0 - 1.4) | (ref) | (ref) |
| Anemia grade 1 | 9,308 | 383 | 25,558 | 1.5 (1.4 - 1.7) | 1.19 (1.02 - 1.39) | 1.05 (0.90 - 1.23) |
| Anemia grade 2 | 8,306 | 322 | 17,175 | 1.9 (1.7 - 2.1) | 1.42 (1.19 - 1.71) | 1.15 (0.94 - 1.40) |
| Anemia grade 3+ | 3,436 | 107 | 5,603 | 1.9 (1.6 - 2.3) | 1.40 (1.09 - 1.78) | 1.07 (0.82 - 1.39) |
| **Fatal stroke** |  |  |  |  |  |  |
| No anemia | 3,866 | 88 | 12,939 | 0.7 (0.5 - 0.8) | (ref) | (ref) |
| Anemia grade 1 | 9,308 | 180 | 26,326 | 0.7 (0.6 - 0.8) | 0.99 (0.82 - 1.21) | 0.91 (0.74 - 1.11) |
| Anemia grade 2 | 8,306 | 169 | 17,760 | 1.0 (0.8 - 1.1) | 1.35 (1.06 - 1.71) | 1.17 (0.90 - 1.52) |
| Anemia grade 3+ | 3,436 | 59 | 5,770 | 1.0 (0.8 - 1.3) | 1.41 (1.02 - 1.95) | 1.28 (0.90 - 1.84) |
| **Nonfatal stroke** |  |  |  |  |  |  |
| No anemia | 3,866 | 180 | 12,619 | 1.4 (1.2 - 1.7) | (ref) | (ref) |
| Anemia grade 1 | 9,308 | 398 | 25,618 | 1.6 (1.4 - 1.7) | 1.06 (0.92 - 1.22) | 0.97 (0.83 - 1.12) |
| Anemia grade 2 | 8,306 | 306 | 17,242 | 1.8 (1.6 - 2.0) | 1.17 (0.99 - 1.39) | 0.96 (0.80 - 1.16) |
| Anemia grade 3+ | 3,436 | 105 | 5,584 | 1.9 (1.5 - 2.3) | 1.20 (0.95 - 1.52) | 0.93 (0.72 - 1.20) |
| **Fatal heart failure** |  |  |  |  |  |  |
| No anemia | 3,866 | 181 | 12,939 | 1.4 (1.2 - 1.6) | (ref) | (ref) |
| Anemia grade 1 | 9,308 | 556 | 26,326 | 2.1 (1.9 - 2.3) | 1.48 (1.28 - 1.70) | 1.19 (1.03 - 1.38) |
| Anemia grade 2 | 8,306 | 539 | 17,760 | 3.0 (2.8 - 3.3) | 2.05 (1.75 - 2.40) | 1.40 (1.18 - 1.66) |
| Anemia grade 3+ | 3,436 | 206 | 5,770 | 3.6 (3.1 - 4.1) | 2.32 (1.91 - 2.83) | 1.48 (1.19 - 1.83) |
| **Nonfatal heart failure** |  |  |  |  |  |  |
| No anemia | 3,866 | 459 | 12,150 | 3.8 (3.4 - 4.1) | (ref) | (ref) |
| Anemia grade 1 | 9,308 | 1,241 | 24,206 | 5.1 (4.8 - 5.4) | 1.29 (1.19 - 1.41) | 1.11 (1.01 - 1.21) |
| Anemia grade 2 | 8,306 | 1,048 | 16,161 | 6.5 (6.1 - 6.9) | 1.52 (1.38 - 1.68) | 1.14 (1.02 - 1.27) |
| Anemia grade 3+ | 3,436 | 432 | 5,163 | 8.4 (7.6 - 9.2) | 1.85 (1.63 - 2.10) | 1.24 (1.08 - 1.43) |
| **Nonfatal unstable angina pectoris** |  |  |  |  |  |  |
| No anemia | 3,866 | 36 | 12,829 | 0.3 (0.2 - 0.4) | (ref) | (ref) |
| Anemia grade 1 | 9,308 | 77 | 26,142 | 0.3 (0.2 - 0.4) | 1.03 (0.75 - 1.40) | 0.89 (0.64 - 1.24) |
| Anemia grade 2 | 8,306 | 47 | 17,661 | 0.3 (0.2 - 0.4) | 0.90 (0.59 - 1.36) | 0.69 (0.43 - 1.11) |
| Anemia grade 3+ | 3,436 | 15 | 5,733 | 0.3 (0.1 - 0.4) | 0.86 (0.47 - 1.57) | 0.58 (0.29 - 1.16) |

^a^ Adjusted for age, gender, marital status, CV history, alcoholism, other comorbidities, recent acute hospitalizations, eGFR level and CKD duration.

**Supplementary Table 4** Crude Incidence rate as well as crude and adjusted^a^ hazard ratios for any and specific fatal and nonfatal cardiovascular events associated with different anemia grades among dialysis dependent patients.

|  | **N** | **No. of events** | **Follow-up time, years** | **Crude incidence rate per 100 person-years (95% CI)** | **Crude hazard ratio (95% CI)** | **Adjusted hazard ratio (95% CI)^a^** |
| --- | --- | --- | --- | --- | --- | --- |
| **Fatal cardiovascular events (CVE)** |  |  |  |  |  |  |
| No anemia | 239 | 13 | 1,300 | 1.0 (0.5 - 1.7) | (ref) | (ref) |
| Anemia grade 1 | 725 | 65 | 3,293 | 2.0 (1.5 - 2.5) | 1.90 (1.12 - 3.21) | 1.41 (0.82 - 2.41) |
| Anemia grade 2 | 1,326 | 133 | 4,476 | 3.0 (2.5 - 3.5) | 2.68 (1.55 - 4.65) | 1.76 (0.99 - 3.15) |
| Anemia grade 3+ | 1,304 | 98 | 3,060 | 3.2 (2.6 - 3.9) | 2.68 (1.50 - 4.78) | 1.84 (0.99 - 3.43) |
| **Nonfatal CVE** |  |  |  |  |  |  |
| No anemia | 239 | 40 | 1,221 | 3.3 (2.3 - 4.5) | (ref) | (ref) |
| Anemia grade 1 | 725 | 144 | 2,941 | 4.9 (4.1 - 5.8) | 1.39 (1.02 - 1.89) | 0.99 (0.72 - 1.35) |
| Anemia grade 2 | 1,326 | 288 | 3,898 | 7.4 (6.6 - 8.3) | 1.91 (1.40 - 2.61) | 1.12 (0.80 - 1.56) |
| Anemia grade 3+ | 1,304 | 227 | 2,702 | 8.4 (7.3 - 9.6) | 1.99 (1.43 - 2.76) | 1.15 (0.80 - 1.66) |
| **Fatal myocardial infarction** |  |  |  |  |  |  |
| No anemia^b^ | 239 | 3 | 1,300 | 0.2 (0.0 - 0.7) | - | - |
| Anemia grade 1 | 725 | 22 | 3,293 | 0.7 (0.4 - 1.0) | (ref) | (ref) |
| Anemia grade 2 | 1,326 | 29 | 4,476 | 0.6 (0.4 - 0.9) | 0.86 (0.57 - 1.31) | 0.70 (0.46 - 1.09) |
| Anemia grade 3+ | 1,304 | 17 | 3,060 | 0.6 (0.3 - 0.9) | 0.66 (0.37 - 1.18) | 0.59 (0.32 - 1.10) |
| **Nonfatal myocardial infarction** |  |  |  |  |  |  |
| No anemia^b^ | 239 | 8 | 1,284 | 0.6 (0.3 - 1.2) | - | - |
| Anemia grade 1 | 725 | 45 | 3,200 | 1.4 (1.0 - 1.9) | (ref) | (ref) |
| Anemia grade 2 | 1,326 | 77 | 4,336 | 1.8 (1.4 - 2.2) | 1.16 (0.90 - 1.52) | 0.98 (0.74 - 1.28) |
| Anemia grade 3+ | 1,304 | 51 | 2,975 | 1.7 (1.3 - 2.3) | 1.03 (0.70 - 1.51) | 0.88 (0.58 - 1.33) |
| **Fatal stroke** |  |  |  |  |  |  |
| No anemia^b^ | 239 | 4 | 1,300 | 0.3 (0.1 - 0.8) | - | - |
| Anemia grade 1 | 725 | 16 | 3,293 | 0.5 (0.3 - 0.8) | (ref) | (ref) |
| Anemia grade 2 | 1,326 | 38 | 4,476 | 0.8 (0.6 - 1.2) | 1.65 (1.05 - 2.58) | 1.49 (0.95 - 2.34) |
| Anemia grade 3+ | 1,304 | 30 | 3,060 | 1.0 (0.7 - 1.4) | 1.80 (1.08 - 3.01) | 1.59 (0.93 - 2.75) |
| **Nonfatal stroke** |  |  |  |  |  |  |
| No anemia | 239 | 15 | 1,266 | 1.2 (0.7 - 2.0) | (ref) | (ref) |
| Anemia grade 1 | 725 | 51 | 3,158 | 1.6 (1.2 - 2.1) | 1.30 (0.80 - 2.12) | 0.93 (0.58 - 1.48) |
| Anemia grade 2 | 1,326 | 95 | 4,270 | 2.2 (1.8 - 2.7) | 1.68 (1.02 - 2.74) | 1.05 (0.63 - 1.74) |
| Anemia grade 3+ | 1,304 | 66 | 2,941 | 2.2 (1.7 - 2.9) | 1.58 (0.93 - 2.69) | 1.01 (0.58 - 1.78) |
| **Fatal heart failure** |  |  |  |  |  |  |
| No anemia^b^ | 239 | 7 | 1,300 | 0.5 (0.2 - 1.1) | - | - |
| Anemia grade 1 | 725 | 32 | 3,293 | 1.0 (0.7 - 1.4) | (ref) | (ref) |
| Anemia grade 2 | 1,326 | 72 | 4,476 | 1.6 (1.3 - 2.0) | 1.57 (1.15 - 2.14) | 1.41 (1.04 - 1.91) |
| Anemia grade 3+ | 1,304 | 56 | 3,060 | 1.8 (1.4 - 2.4) | 1.65 (1.13 - 2.42) | 1.58 (1.06 - 2.37) |
| **Nonfatal heart failure** |  |  |  |  |  |  |
| No anemia | 239 | 21 | 1,264 | 1.7 (1.0 - 2.5) | (ref) | (ref) |
| Anemia grade 1 | 725 | 82 | 3,125 | 2.6 (2.1 - 3.3) | 1.48 (0.98 - 2.24) | 1.24 (0.80 - 1.91) |
| Anemia grade 2 | 1,326 | 168 | 4,190 | 4.0 (3.4 - 4.7) | 2.06 (1.34 - 3.19) | 1.50 (0.94 - 2.41) |
| Anemia grade 3+ | 1,304 | 140 | 2,891 | 4.8 (4.1 - 5.7) | 2.28 (1.44 - 3.61) | 1.67 (1.00 - 2.78) |
| **Nonfatal unstable angina pectoris** |  |  |  |  |  |  |
| No anemia^b^ | 239 | 1 | 1,299 | 0.1 (0.0 - 0.4) | - | - |
| Anemia grade 1 | 725 | 14 | 3,243 | 0.4 (0.2 - 0.7) | (ref) | (ref) |
| Anemia grade 2 | 1,326 | 24 | 4,399 | 0.5 (0.3 - 0.8) | 1.14 (0.74 - 1.75) | 0.92 (0.57 - 1.48) |
| Anemia grade 3+ | 1,304 | 15 | 3,014 | 0.5 (0.3 - 0.8) | 0.94 (0.49 - 1.80) | 0.72 (0.34 - 1.52) |

^a^Adjusted for age, gender, marital status, CV history, alcoholism, other comorbidities, recent acute hospitalizations, eGFR level and CKD duration.

**Supplementary Table 5**

Crude and adjusted^a^ hazard ratios for incident dialysis, all cause death, any and specific cardiovascular events stratified by fatal and non-fatal events associated with different anemia grades among non-dialysis dependent severe CKD patients split by follow up time (0-2 years and 2-8 years).

|  |  | **Follow-up** | **Crude hazard ratio (95% CI)** | **Adjusted hazard ratio (95% CI)^a^** |
| --- | --- | --- | --- | --- |
| Incident dialysis |  |  |  |  |
|  | Anemia grade 1 | 0-2 years | 2.88 (2.36 - 3.51) | 2.25 (1.84 - 2.75) |
|  | Anemia grade 1 | 2-8 years | 1.25 (1.09 - 1.44) | 0.93 (0.80 - 1.08) |
|  | Anemia grade 2 | 0-2 years | 5.99 (4.86 - 7.39) | 4.05 (3.24 - 5.05) |
|  | Anemia grade 2 | 2-8 years | 1.21 (1.01 - 1.46) | 0.81 (0.67 - 0.99) |
|  | Anemia grade 3+ | 0-2 years | 7.59 (6.02 - 9.56) | 4.79 (3.71 - 6.17) |
|  | Anemia grade 3+ | 2-8 years | 1.25 (0.95 - 1.65) | 0.80 (0.59 - 1.08) |
| Acute hospitalization |  |  |  |  |
|  | Anemia grade 1 | 0-2 years | 1.36 (1.28 - 1.45) | 1.22 (1.14 - 1.30) |
|  | Anemia grade 1 | 2-8 years | 1.13 (1.03 - 1.23) | 1.03 (0.94 - 1.12) |
|  | Anemia grade 2 | 0-2 years | 2.15 (2.00 - 2.32) | 1.66 (1.53 - 1.80) |
|  | Anemia grade 2 | 2-8 years | 1.34 (1.18 - 1.52) | 1.06 (0.93 - 1.21) |
|  | Anemia grade 3+ | 0-2 years | 3.05 (2.71 - 3.44) | 2.16 (1.90 - 2.45) |
|  | Anemia grade 3+ | 2-8 years | 1.54 (1.23 - 1.93) | 1.11 (0.87 - 1.41) |
| All-cause death |  |  |  |  |
|  | Anemia grade 1 | 0-2 years | 1.41 (1.32 - 1.51) | 1.22 (1.14 - 1.31) |
|  | Anemia grade 1 | 2-8 years | 1.22 (1.14 - 1.30) | 1.11 (1.04 - 1.18) |
|  | Anemia grade 2 | 0-2 years | 2.31 (2.15 - 2.48) | 1.76 (1.63 - 1.90) |
|  | Anemia grade 2 | 2-8 years | 1.52 (1.41 - 1.65) | 1.27 (1.17 - 1.37) |
|  | Anemia grade 3+ | 0-2 years | 3.16 (2.90 - 3.43) | 2.38 (2.17 - 2.61) |
|  | Anemia grade 3+ | 2-8 years | 1.59 (1.43 - 1.77) | 1.34 (1.19 - 1.50) |
| Cardiovascular events (CVE) |  |  |  |  |
|  | Anemia grade 1 | 0-2 years | 1.32 (1.21 - 1.44) | 1.13 (1.04 - 1.24) |
|  | Anemia grade 1 | 2-8 years | 1.15 (1.02 - 1.28) | 1.02 (0.91 - 1.15) |
|  | Anemia grade 2 | 0-2 years | 1.67 (1.52 - 1.84) | 1.24 (1.12 - 1.38) |
|  | Anemia grade 2 | 2-8 years | 1.22 (1.07 - 1.40) | 0.97 (0.85 - 1.12) |
|  | Anemia grade 3+ | 0-2 years | 1.86 (1.65 - 2.10) | 1.27 (1.11 - 1.45) |
|  | Anemia grade 3+ | 2-8 years | 1.59 (1.32 - 1.92) | 1.14 (0.94 - 1.38) |
| Fatal CVE |  |  |  |  |
|  | Anemia grade 1 | 0-2 years | 1.37 (1.18 - 1.60) | 1.13 (0.97 - 1.32) |
|  | Anemia grade 1 | 2-8 years | 1.36 (1.14 - 1.61) | 1.17 (0.99 - 1.40) |
|  | Anemia grade 2 | 0-2 years | 1.93 (1.63 - 2.28) | 1.39 (1.16 - 1.65) |
|  | Anemia grade 2 | 2-8 years | 1.89 (1.55 - 2.31) | 1.47 (1.20 - 1.81) |
|  | Anemia grade 3+ | 0-2 years | 2.16 (1.76 - 2.65) | 1.49 (1.20 - 1.85) |
|  | Anemia grade 3+ | 2-8 years | 2.18 (1.67 - 2.84) | 1.67 (1.27 - 2.20) |
| Nonfatal CVE |  |  |  |  |
|  | Anemia grade 1 | 0-2 years | 1.30 (1.18 - 1.44) | 1.12 (1.02 - 1.24) |
|  | Anemia grade 1 | 2-8 years | 1.13 (1.00 - 1.28) | 1.00 (0.89 - 1.14) |
|  | Anemia grade 2 | 0-2 years | 1.67 (1.50 - 1.87) | 1.26 (1.12 - 1.41) |
|  | Anemia grade 2 | 2-8 years | 1.13 (0.97 - 1.31) | 0.90 (0.77 - 1.05) |
|  | Anemia grade 3+ | 0-2 years | 1.91 (1.67 - 2.19) | 1.30 (1.12 - 1.50) |
|  | Anemia grade 3+ | 2-8 years | 1.44 (1.17 - 1.78) | 1.01 (0.81 - 1.26) |
| Myocardial infarct |  |  |  |  |
|  | Anemia grade 1 | 0-2 years | 1.37 (0.99 - 1.89) | 1.18 (0.85 - 1.63) |
|  | Anemia grade 1 | 2-8 years | 1.40 (0.94 - 2.09) | 1.27 (0.86 - 1.89) |
|  | Anemia grade 2 | 0-2 years | 1.74 (1.21 - 2.51) | 1.41 (0.97 - 2.07) |
|  | Anemia grade 2 | 2-8 years | 1.79 (1.11 - 2.90) | 1.57 (0.97 - 2.56) |
|  | Anemia grade 3+ | 0-2 years | 1.45 (0.89 - 2.35) | 1.21 (0.73 - 2.01) |
|  | Anemia grade 3+ | 2-8 years | 2.47 (1.36 - 4.47) | 2.31 (1.25 - 4.28) |
| Fatal myocardial infarct |  |  |  |  |
|  | Anemia grade 1 | 0-2 years | 1.30 (0.87 - 1.94) | 1.12 (0.75 - 1.69) |
|  | Anemia grade 1 | 2-8 years | 1.40 (0.86 - 2.28) | 1.26 (0.77 - 2.07) |
|  | Anemia grade 2 | 0-2 years | 1.59 (1.07 - 2.35) | 1.26 (0.84 - 1.90) |
|  | Anemia grade 2 | 2-8 years | 1.79 (1.09 - 2.95) | 1.53 (0.92 - 2.55) |
|  | Anemia grade 3+ | 0-2 years | 1.27 (0.75 - 2.15) | 1.03 (0.60 - 1.78) |
|  | Anemia grade 3+ | 2-8 years | 2.46 (1.32 - 4.58) | 2.20 (1.15 - 4.22) |
| Nonfatal myocardial infarct |  |  |  |  |
|  | Anemia grade 1 | 0-2 years | 1.21 (0.98 - 1.51) | 1.07 (0.86 - 1.33) |
|  | Anemia grade 1 | 2-8 years | 1.22 (0.95 - 1.58) | 1.08 (0.83 - 1.39) |
|  | Anemia grade 2 | 0-2 years | 1.69 (1.33 - 2.13) | 1.37 (1.06 - 1.76) |
|  | Anemia grade 2 | 2-8 years | 1.16 (0.85 - 1.58) | 0.95 (0.69 - 1.31) |
|  | Anemia grade 3+ | 0-2 years | 1.60 (1.19 - 2.16) | 1.23 (0.90 - 1.70) |
|  | Anemia grade 3+ | 2-8 years | 1.34 (0.86 - 2.06) | 1.06 (0.68 - 1.64) |
| Stroke |  |  |  |  |
|  | Anemia grade 1 | 0-2 years | 1.10 (0.93 - 1.30) | 1.00 (0.84 - 1.18) |
|  | Anemia grade 1 | 2-8 years | 0.98 (0.80 - 1.20) | 0.90 (0.74 - 1.10) |
|  | Anemia grade 2 | 0-2 years | 1.32 (1.09 - 1.60) | 1.09 (0.89 - 1.34) |
|  | Anemia grade 2 | 2-8 years | 1.07 (0.84 - 1.35) | 0.91 (0.71 - 1.16) |
|  | Anemia grade 3+ | 0-2 years | 1.41 (1.10 - 1.81) | 1.13 (0.86 - 1.48) |
|  | Anemia grade 3+ | 2-8 years | 1.19 (0.83 - 1.68) | 0.99 (0.69 - 1.44) |
| Fatal stroke |  |  |  |  |
|  | Anemia grade 1 | 0-2 years | 1.11 (0.82 - 1.50) | 0.99 (0.73 - 1.34) |
|  | Anemia grade 1 | 2-8 years | 0.90 (0.66 - 1.24) | 0.83 (0.60 - 1.14) |
|  | Anemia grade 2 | 0-2 years | 1.56 (1.11 - 2.18) | 1.28 (0.90 - 1.84) |
|  | Anemia grade 2 | 2-8 years | 1.25 (0.86 - 1.80) | 1.10 (0.75 - 1.62) |
|  | Anemia grade 3+ | 0-2 years | 1.74 (1.14 - 2.66) | 1.49 (0.94 - 2.36) |
|  | Anemia grade 3+ | 2-8 years | 1.22 (0.70 - 2.14) | 1.16 (0.64 - 2.08) |
| Nonfatal stroke |  |  |  |  |
|  | Anemia grade 1 | 0-2 years | 1.12 (0.91 - 1.37) | 1.01 (0.82 - 1.25) |
|  | Anemia grade 1 | 2-8 years | 1.03 (0.82 - 1.30) | 0.93 (0.74 - 1.18) |
|  | Anemia grade 2 | 0-2 years | 1.34 (1.07 - 1.69) | 1.10 (0.85 - 1.41) |
|  | Anemia grade 2 | 2-8 years | 1.03 (0.78 - 1.36) | 0.85 (0.64 - 1.14) |
|  | Anemia grade 3+ | 0-2 years | 1.40 (1.03 - 1.89) | 1.07 (0.77 - 1.48) |
|  | Anemia grade 3+ | 2-8 years | 1.11 (0.73 - 1.68) | 0.87 (0.56 - 1.34) |
| Chronic heart faliure |  |  |  |  |
|  | Anemia grade 1 | 0-2 years | 1.39 (1.26 - 1.54) | 1.16 (1.05 - 1.29) |
|  | Anemia grade 1 | 2-8 years | 1.23 (1.07 - 1.42) | 1.09 (0.94 - 1.26) |
|  | Anemia grade 2 | 0-2 years | 1.80 (1.60 - 2.02) | 1.29 (1.14 - 1.46) |
|  | Anemia grade 2 | 2-8 years | 1.32 (1.11 - 1.57) | 1.03 (0.86 - 1.23) |
|  | Anemia grade 3+ | 0-2 years | 2.13 (1.86 - 2.46) | 1.40 (1.19 - 1.64) |
|  | Anemia grade 3+ | 2-8 years | 1.78 (1.42 - 2.25) | 1.26 (0.99 - 1.60) |
| Fatal heart failure |  |  |  |  |
|  | Anemia grade 1 | 0-2 years | 1.40 (1.16 - 1.69) | 1.11 (0.92 - 1.34) |
|  | Anemia grade 1 | 2-8 years | 1.66 (1.33 - 2.08) | 1.39 (1.11 - 1.75) |
|  | Anemia grade 2 | 0-2 years | 2.01 (1.63 - 2.47) | 1.35 (1.09 - 1.68) |
|  | Anemia grade 2 | 2-8 years | 2.34 (1.79 - 3.04) | 1.71 (1.31 - 2.24) |
|  | Anemia grade 3+ | 0-2 years | 2.36 (1.85 - 3.02) | 1.48 (1.14 - 1.93) |
|  | Anemia grade 3+ | 2-8 years | 2.68 (1.91 - 3.76) | 1.89 (1.33 - 2.68) |
| Nonfatal heart failure |  |  |  |  |
|  | Anemia grade 1 | 0-2 years | 1.40 (1.24 - 1.57) | 1.18 (1.05 - 1.32) |
|  | Anemia grade 1 | 2-8 years | 1.20 (1.02 - 1.40) | 1.06 (0.90 - 1.24) |
|  | Anemia grade 2 | 0-2 years | 1.79 (1.57 - 2.05) | 1.30 (1.13 - 1.50) |
|  | Anemia grade 2 | 2-8 years | 1.24 (1.03 - 1.50) | 0.97 (0.80 - 1.18) |
|  | Anemia grade 3+ | 0-2 years | 2.19 (1.87 - 2.56) | 1.43 (1.20 - 1.71) |
|  | Anemia grade 3+ | 2-8 years | 1.66 (1.29 - 2.14) | 1.16 (0.90 - 1.51) |
| Unstable angina pectoris |  |  |  |  |
|  | Anemia grade 1 | 0-2 years | 1.05 (0.69 - 1.61) | 0.93 (0.61 - 1.44) |
|  | Anemia grade 1 | 2-8 years | 0.98 (0.62 - 1.57) | 0.88 (0.54 - 1.42) |
|  | Anemia grade 2 | 0-2 years | 1.15 (0.69 - 1.91) | 0.92 (0.54 - 1.59) |
|  | Anemia grade 2 | 2-8 years | 0.71 (0.37 - 1.38) | 0.59 (0.29 - 1.21) |
|  | Anemia grade 3+ | 0-2 years | 1.19 (0.61 - 2.30) | 0.86 (0.41 - 1.80) |
|  | Anemia grade 3+ | 2-8 years | 0.91 (0.36 - 2.27) | 0.68 (0.25 - 1.82) |
| Nonfatal unstable angina pectoris |  |  |  |  |
|  | Anemia grade 1 | 0-2 years | 1.05 (0.66 - 1.67) | 0.93 (0.58 - 1.50) |
|  | Anemia grade 1 | 2-8 years | 1.03 (0.63 - 1.68) | 0.90 (0.55 - 1.49) |
|  | Anemia grade 2 | 0-2 years | 1.08 (0.63 - 1.87) | 0.87 (0.49 - 1.57) |
|  | Anemia grade 2 | 2-8 years | 0.74 (0.37 - 1.47) | 0.60 (0.28 - 1.25) |
|  | Anemia grade 3+ | 0-2 years | 1.01 (0.48 - 2.14) | 0.73 (0.32 - 1.68) |
|  | Anemia grade 3+ | 2-8 years | 0.78 (0.28 - 2.17) | 0.56 (0.19 - 1.65) |

^a^ Adjusted for age, gender, marital status, CV history, alcoholism, other comorbidities, recent acute hospitalizations, eGFR level and CKD duration.

**Supplementary Table 6**

Crude and adjusted^a^ hazard ratios for all-cause death, any and specific cardiovascular events associated with different anemia grades among dialysis dependent patients stratified by follow up time (0-2 years and 2-8 years)

|  |  | **Follow-up** | **Crude hazard ratio (95% CI)** | **Adjusted hazard ratio (95% CI)** |
| --- | --- | --- | --- | --- |
| Acute hospitalization |  |  |  |  |
|  | Anemia grade 1 | 0-2 years | 2.05 (1.61 - 2.61) | 1.20 (0.92 - 1.56) |
|  | Anemia grade 1 | 2-8 years | 1.49 (1.12 - 1.98) | 1.01 (0.76 - 1.34) |
|  | Anemia grade 2 | 0-2 years | 3.31 (2.58 - 4.24) | 1.44 (1.08 - 1.92) |
|  | Anemia grade 2 | 2-8 years | 1.59 (1.16 - 2.18) | 0.83 (0.60 - 1.16) |
|  | Anemia grade 3+ | 0-2 years | 4.61 (3.52 - 6.03) | 1.99 (1.46 - 2.71) |
|  | Anemia grade 3+ | 2-8 years | 1.04 (0.65 - 1.67) | 0.51 (0.30 - 0.86) |
| All-cause death |  |  |  |  |
|  | Anemia grade 1 | 0-2 years | 1.71 (1.22 - 2.39) | 1.25 (0.87 - 1.79) |
|  | Anemia grade 1 | 2-8 years | 1.81 (1.39 - 2.36) | 1.27 (0.95 - 1.68) |
|  | Anemia grade 2 | 0-2 years | 2.68 (1.89 - 3.80) | 1.66 (1.14 - 2.41) |
|  | Anemia grade 2 | 2-8 years | 2.31 (1.74 - 3.05) | 1.37 (1.01 - 1.85) |
|  | Anemia grade 3+ | 0-2 years | 4.15 (2.90 - 5.93) | 2.46 (1.67 - 3.62) |
|  | Anemia grade 3+ | 2-8 years | 2.17 (1.60 - 2.95) | 1.38 (0.99 - 1.92) |
| Cardiovascular events |  |  |  |  |
|  | Anemia grade 1 | 0-2 years | 1.46 (0.96 - 2.22) | 0.99 (0.64 - 1.54) |
|  | Anemia grade 1 | 2-8 years | 1.48 (1.00 - 2.19) | 1.05 (0.71 - 1.54) |
|  | Anemia grade 2 | 0-2 years | 2.16 (1.42 - 3.26) | 1.23 (0.78 - 1.92) |
|  | Anemia grade 2 | 2-8 years | 1.86 (1.23 - 2.81) | 1.04 (0.69 - 1.57) |
|  | Anemia grade 3+ | 0-2 years | 2.50 (1.63 - 3.83) | 1.40 (0.87 - 2.23) |
|  | Anemia grade 3+ | 2-8 years | 1.47 (0.92 - 2.36) | 0.84 (0.53 - 1.35) |
| Myocardial infarction |  |  |  |  |
|  | Anemia grade 1 | 0-2 years | 2.64 (1.11 - 6.29) | 1.35 (0.54 - 3.39) |
|  | Anemia grade 1 | 2-8 years | 1.93 (0.89 - 4.22) | 0.85 (0.38 - 1.87) |
|  | Anemia grade 2 | 0-2 years | 2.75 (1.11 - 6.79) | 1.14 (0.43 - 3.02) |
|  | Anemia grade 2 | 2-8 years | 2.36 (1.00 - 5.53) | 0.80 (0.33 - 1.93) |
|  | Anemia grade 3+ | 0-2 years | 2.99 (1.19 - 7.55) | 1.23 (0.45 - 3.39) |
|  | Anemia grade 3+ | 2-8 years | 1.30 (0.47 - 3.58) | 0.47 (0.17 - 1.30) |
| Stroke |  |  |  |  |
|  | Anemia grade 1 | 0-2 years | 1.84 (0.80 - 4.21) | 1.27 (0.55 - 2.94) |
|  | Anemia grade 1 | 2-8 years | 1.03 (0.60 - 1.76) | 0.68 (0.41 - 1.13) |
|  | Anemia grade 2 | 0-2 years | 2.67 (1.19 - 5.97) | 1.62 (0.71 - 3.69) |
|  | Anemia grade 2 | 2-8 years | 1.28 (0.73 - 2.25) | 0.74 (0.42 - 1.28) |
|  | Anemia grade 3+ | 0-2 years | 2.78 (1.21 - 6.38) | 1.67 (0.70 - 3.98) |
|  | Anemia grade 3+ | 2-8 years | 1.19 (0.62 - 2.27) | 0.73 (0.39 - 1.37) |
| Heart failure |  |  |  |  |
|  | Anemia grade 1 | 0-2 years | 1.15 (0.68 - 1.96) | 0.93 (0.54 - 1.61) |
|  | Anemia grade 1 | 2-8 years | 1.75 (1.03 - 2.98) | 1.50 (0.86 - 2.60) |
|  | Anemia grade 2 | 0-2 years | 2.01 (1.18 - 3.42) | 1.43 (0.81 - 2.51) |
|  | Anemia grade 2 | 2-8 years | 2.12 (1.18 - 3.78) | 1.49 (0.82 - 2.71) |
|  | Anemia grade 3+ | 0-2 years | 2.47 (1.44 - 4.25) | 1.76 (0.98 - 3.15) |
|  | Anemia grade 3+ | 2-8 years | 1.60 (0.82 - 3.14) | 1.18 (0.59 - 2.35) |

^a^ Adjusted for age, gender, marital status, CV history, alcoholism, other comorbidities, recent acute hospitalizations, eGFR level and CKD duration.
